# Osseous Resective Surgery: The Past, the Present and the Future

**DOI:** 10.1111/jre.70029

**Published:** 2025-09-02

**Authors:** Mario Aimetti, Gianfranco Carnevale

**Affiliations:** ^1^ Department of Periodontology, University of Torino; ^2^ Private Practice in Rome

## 1 Introduction

The main goal of periodontal therapy is to arrest the destructive progression of disease and preserve natural dentition. The resolution of inflammation, the reduction of pocket depth (PD), and the stabilization or improvement of clinical attachment level (CAL) are the core objectives of treatment. These therapeutic outcomes are typically pursued through a stepwise approach beginning with risk factor control and non‐surgical therapy (Step I and II) [1]. Ideally, these early interventions lead to a clinical scenario characterized by probing depths of ≤ 4 mm, a biologically favorable condition that enables long‐term periodontal stability [2, 3]. However, in many patients, pockets ≥ 4 mm with bleeding on probing persist despite comprehensive non‐surgical therapy [4, 5]. These sites represent an ongoing risk for disease progression and typically warrant surgical intervention to achieve a more stable periodontal environment [6].

Periodontal therapy offers three distinct histological outcomes: repair, most commonly characterized by the formation of a long junctional epithelium; regeneration, involving the restoration of periodontal ligament, cementum, and alveolar bone; and what might be termed a “reset”—the reestablishment of a physiologic situation with a short junctional epithelium and a minimal probing depth, achieved through apical positioning of the marginal periodontal tissues [7]. Each of these outcomes carries different biological and clinical implications, and the chosen surgical strategy should take into account the patient‐specific risk profile, reflect the anatomy of the defect, and aim to achieve predictable long‐term therapeutic outcomes.

Among available surgical modalities, flap surgery with osseous resection (ORS) remains a well‐established and predictable technique, specifically aimed at eliminating periodontal pockets, reestablishing a “correct” and maintainable anatomical architecture, and preserving or increasing an adequate band of attached gingiva [8, 9]. Despite the concept—supported by controlled clinical trials—that long‐term outcomes of different periodontal surgical procedures may converge, ORS continues to represent a highly effective and predictable clinical tool. Its goal is not only to eliminate pockets but also to correct soft and hard tissue deformities, thereby restoring a functional dento‐alveolar relationship conducive to long‐term stability. When properly indicated and meticulously executed, ORS contributes meaningfully to periodontal health and remains one of the principal surgical strategies with documented long‐term success. Importantly, ORS achieves the anatomical, histological, and physiological outcome of a healthy periodontium albeit positioned at a more apical level. This deliberate architectural reset creates a new, stable marginal profile that facilitates effective plaque control and reduces the risk of disease recurrence when combined with appropriate supportive periodontal therapy.

## 2 The Past

The removal of superficial radicular and interproximal alveolar bone has been part of periodontal therapy for more than a century. Early practitioners believed that alveolar bone was necrotic and required removal. Over time, this understanding evolved. Pioneers such as Widman and Neumann began reshaping the alveolar bone to support flap adaptation and reestablish physiologic anatomy. Carranza Sr. contributed early insights into the recontouring of bone to guide gingival tissue toward a more physiological position. Schluger's seminal 1949 article laid out the principles of osseous surgery to reestablish a positive bone architecture conducive to predictable pocket elimination—a concept later popularized by Prichard, Friedman, and Ochsenbein [10, 11, 12].

Subsequent decades brought comparative clinical trials examining the short‐ and long‐term efficacy of ORS relative to other periodontal therapies. In a split‐mouth study, Knowles et al. reported that in 4–6 mm pockets, ORS achieved greater reduction in probing depth at 5 years compared to curettage, with results comparable to modified Widman flap surgery. Although the gain in CAL was similar across techniques, the durability of PD reduction supported the efficacy of ORS. In deeper pockets (≥ 7 mm), the three therapies yielded equivalent reductions in probing depth and CAL gains [13]. Again, from the Michigan school, Ramfjord et al. evaluated 72 patients undergoing root planing, modified Widman, or ORS. After 5 years of SPC, reductions in probing depth were broadly comparable across all groups. However, the probing methodology was referenced in a prior publication and remains unclear, particularly regarding how interproximal sites were assessed [14]. This detail is clinically relevant, as Rosenberg later emphasized: interproximal areas are typically where periodontal breakdown is most severe, and where recurrence of disease most frequently occurs [15]. Despite this limitation, the average depth of pocket sites treated with ORS exhibited half the loss of gained attachment compared to the other groups. Notably, molar furcation sites were excluded from the analysis [16].

From Nebraska, Kaldahl et al. conducted one of the most important clinical studies of pocket therapy; a comprehensive five‐year comparison of the three main therapeutic modalities. In 5–6 mm pockets, ORS achieved a significantly greater PD reduction than modified Widman and root planing. In ≥ 7 mm pockets, ORS again demonstrated superior PD reduction, with similar CAL outcomes across all three treatments [17]. Interpretation of these studies is nuanced.

Detailed intrasurgical measurements—such as the anatomy and depth of intrabony or hemiseptal defects, surgical correction, or flap positioning—were often missing. Without this information, distinguishing cases where ORS was ideally indicated becomes difficult. An 8‐mm pocket, for instance, may reflect a 2 or a 5‐mm intrabony component, with vastly different surgical implications. As such, clinical context and defect morphology are essential in evaluating the appropriateness and effectiveness of ORS.

Importantly, longitudinal data demonstrated that sites treated with ORS exhibit a significantly lower incidence of clinical attachment loss over time. In a seven‐year follow‐up study, ORS‐treated sites experienced fewer episodes of ≥ 3 mm attachment loss compared to those treated with root planing or modified Widman surgery [18]. This lower rate of disease recurrence may be attributed to the greater pocket reduction achieved with this type of surgical therapy, enhancing long‐term maintainability.

Inflammatory indices (e.g., bleeding on probing, gingival index, suppuration) have been found to be similar among surgical and non‐surgical approaches in both short‐ and long‐term studies [19, 20, 21]. Concerns regarding supragingival plaque control following ORS—due to lengthened clinical crowns—have been balanced by evidence that open embrasures promote the effective use of interdental brushes. As such, ORS may enhance self‐performed plaque control rather than impair it [22, 23].

The success of ORS, however, has never been immune to critique. The foundational assumption of the era—that “pocket equals disease”—became a double‐edged sword. While it encouraged therapeutic rigor, it also created a dogma where the presence of a residual pocket, regardless of inflammation or stability, was seen as failure. In this context, surgical pocket elimination became an almost imperative indication.

Critics questioned this view. Beginning in the 1980s, studies on guided tissue regeneration (GTR) and biologically active materials began to shift the focus from pocket elimination to attachment gain [24]. If the goal was not merely to reduce probing depth but to reestablish connective tissue attachment and bone, then resective surgery—with its predictable loss of soft tissue and potential reduction of support—seemed at odds with regenerative ambitions.

The tension between function and esthetics also surfaced. The apical displacement of gingival margins following ORS, while effective in reducing PD, often resulted in increased recession with longer clinical crowns and exposed root surfaces. What had once been considered a clinical success began to be reconsidered in light of esthetic demands and patient comfort, particularly with the advent of root coverage procedures and mucogingival surgery. Gingival recession became not only a technical consequence but a patient‐centered concern.

Moreover, some critics equated ORS with the creation of a “flat” periodontium—a oversimplified architecture that, while easy to clean, may not respect the natural curvature and biologic diversity of the dentogingival complex. In the pursuit of maintainability, anatomical nuance risked being lost. Nonetheless, these critiques also drove evolution. The rigid application of ORS began to evolve toward a more patient‐centered, site‐specific, anatomy‐aware, and tissue‐preserving philosophy.

## 3 The Present

Today, apically positioned flap and ORS remain a keystone of the periodontal surgical repertoire, particularly for the management of suprabony and shallow intrabony defects where pocket elimination and long‐term access for maintenance are the primary objectives. Its relevance is especially pronounced in posterior regions—areas notoriously challenging for both effective domiciliary plaque control and thorough professional subgingival instrumentation. Its effectiveness, however, depends heavily on careful case selection, technical execution, and precise soft tissue handling. ORS is generally not indicated for deep intrabony lesions where regenerative procedures are favored, and its use in advanced cases often entails a trade‐off: predictable pocket reduction at the expense of some clinical attachment loss in neighboring, less involved sites.

Despite its historical origins, considerable misconceptions persist regarding the surgical execution of ORS. In fact, ORS is not a single act but a coordinated sequence of surgical maneuvers. These include access to the root and alveolar margin, elimination of the gingival pocket, root debridement, correction of bony architecture, and repositioning of the flap to a more apical level. Achieving a maintainable positive architecture requires the combined application of ostectomy and osteoplasty.

A key element in the success of ORS lies in meticulous flap design and management. Apical flap positioning usually requires periosteal anchorage and adequate flap mobility, typically obtained via split‐thickness extension into the alveolar mucosa and vertical‐releasing incisions when needed. On the palatal side, thinning and scalloping of the flap allow adaptation to the underlying osseous profile.

When correctly performed, ORS results in minimal removal of supporting bone. For instance, at the interradicular crest, Moghaddas and Stahl reported that the average bone removed ranged between 0.09–0.12 mm, with facial and lingual aspects averaging around 0.3 mm [25]. Other studies have documented an overall range from 0.6 to 1.2 mm of ostectomy per site [26, 27].

Traditional ORS has typically relied on the most‐apical point of the interdental alveolar crest to guide the amount of interproximal and radicular bone resection necessary to recreate a physiologic profile. However, histological studies have consistently shown that supracrestal connective tissue fibers are retained coronally to the alveolar bone both in health and disease [28, 29]. This raises the question of whether complete removal of these fibers during surgery is necessary—or even beneficial.

In this context, a refined approach to ORS has been introduced by Carnevale: FibReORS (Fiber Retention Osseous Resective Surgery) [30, 31]. This technique preserves supracrestal connective tissue fibers, allowing a more coronal positioning of the base of the defect and reducing the need for supporting bone removal. FibReORS represents a paradigm shift in how interproximal defects are managed, treating the mineralized and unmineralized connective tissue complex as a functional unit.

Randomized clinical trials support the effectiveness of FibReORS [32, 33]. Aimetti and colleagues reported that shallow PDs were maintained over a 12‐ to 48‐month period with minimal clinical complications; ostectomy amounted to 1.0 ± 0.3 mm in the ORS group and to 0.4 ± 0.2 mm in the FibReORS group [33, 34, 35]. At four years, over 91% of FibReORS‐treated sites achieved complete pocket closure, and 95.3% presented with PPD ≤ 3 mm and no bleeding. Importantly, full‐mouth plaque and bleeding scores remained < 15% during maintenance, contributing to the overall stability of outcomes [34].

Soft tissue rebound was then observed in both ORS and FibReORS groups [34, 36]. However, in the FibReORS‐treated sextants, the coronal migration of the gingival margin was largely completed within the first 12 months, while ORS‐treated sites showed a slower rebound extending up to 48 months [37, 38]. The gingival margin rose approximately 2.4–2.5 mm coronally from its postsurgical position in both groups, with greater creeping seen in interproximal areas. Additionally, a mean gain in keratinized tissue width (~2 mm) was reported across the follow‐up period, indicating soft tissue health and periodontal stability.

A 2020 systematic review and meta‐analysis provided robust evidence supporting the clinical efficacy of ORS. At 12 months, the weighted mean percentage of pocket elimination (defined as final PD ≤ 4 mm) was around 98%, with no substantial difference between ORS and FibReORS procedures [39]. Importantly, the mean amount of ostectomy was limited to 0.87 mm, while gingival recession increased by 2.13 mm on average. The fiber retention technique was associated with a reduced biological cost, showing less bone removal (0.40 mm vs. 1.04 mm) and reduced gingival recession (1.72 mm vs. 2.33 mm) compared to conventional ORS.

These findings underscore the relevance of ORS—especially in its modern, tissue‐preserving forms—as a reliable tool for achieving periodontal stability. The approach requires skill, precision, and an understanding of biological principles, but when performed appropriately, the reproducible results are both functionally and biologically sustainable.

## 4 The Future

Looking ahead, the relevance of ORS in modern periodontal therapy hinges not on whether bone is removed, but on why and how. In an era that emphasizes minimally invasive approaches and tissue preservation, the notion of deliberately resecting bone and losing attachment may seem counterintuitive. Yet, when the therapeutic objective is stable, shallow probing depths—particularly in restorative or interdisciplinary cases, even more when implants must be used—ORS offers a direct path to a proper and more favorable environment for teeth and implant health in patients susceptible to periodontal disease. Actually, ORS does not represent overtreatment—but a targeted architectural reset, offering a biologically sound and efficient journey to long‐term stability.

In scenarios where a patient presents with generalized mild‐to‐moderate pockets, conservative options such as re‐instrumentation or access flap procedures aimed at long junctional epithelium formation may suffice. However, in complex cases—especially those requiring restorative margins placed at or near the gingival margin—a surgically established shallow sulcus via ORS may provide the most predictable and stable long‐term outcomes. In such situations, a stable, histological short junctional epithelium contributes not only to disease control but also to the positional stability and shape of the soft tissues and physiological environment.

Concerns about recession, hypersensitivity, or risk of root caries can usually be well controlled through personalized and comprehensive care. The aesthetic compromises associated with ORS are often acceptable, not clinically relevant, or even beneficial in posterior sextants in pre‐prosthetic treatment. When aligned with individualized patient risk profiles, the decision to perform ORS becomes a strategic one—balancing clinical goals, anatomical limitations, and long‐term maintenance potential.

The introduction of digital planning, including 3D imaging and augmented reality in periodontal surgery, also allows for refined visualization of bone topography. Potentially, this enables personalized flap design and more controlled bone recontouring for digitally guided osseous surgery. Future workflows may involve pre‐surgical simulations that define not only how and where to reshape bone in order to obtain a maintainable anatomical condition optimizing the amount of ostectomy but also the ideal flap design to preserve supracrestal soft tissues. Moreover, it may be considered the potentiality of new technologies like piezo surgery. Specifically, piezoelectric inserts may facilitate the application of minimally invasive principles to the ORS approach, enabling precise bone recontouring while preserving fiber attachment.

From a biological standpoint, further integration of ORS with host‐modulatory strategies may improve outcomes. Anti‐inflammatory adjuncts, healing‐promoting agents, and inductors targeting wound healing could redefine the tissue response after resective procedures. With such support, the biological cost of surgery could be further reduced, enhancing both clinical and patient‐centered outcomes.

Ultimately, ORS remains a critical therapeutic arrow in the clinician's quiver. Very important is to underline that no technique is without indications and contraindications, but when the goal is long‐term attachment stability and tooth preservation, osseous resective therapy deserves renewed attention. Contemporary advances—such as Fiber Retention protocols and minimally invasive surgical principles—can enhance both outcomes and patient acceptance, proving that a century‐old technique can still thrive in a modern periodontal practice.

## Conflicts of Interest

The authors declare no conflicts of interest.

## Data Availability

The data that support the findings of this study are available from the corresponding author upon reasonable request.
